# Impaired Luminal Control of Intestinal Macrophage Maturation in Patients With Ulcerative Colitis During Remission

**DOI:** 10.1016/j.jcmgh.2021.06.004

**Published:** 2021-06-12

**Authors:** Lujain Maasfeh, Anetta Härtlova, Stefan Isaksson, Johanna Sundin, Georgios Mavroudis, Otto Savolainen, Hans Strid, Lena Öhman, Maria K. Magnusson

**Affiliations:** 1 Department of Microbiology and Immunology, Institute of Biomedicine, Sahlgrenska Academy, University of Gothenburg, Gothenburg, Sweden; 2 Wallenberg Centre for Molecular and Translational Medicine, Institute of Biomedicine, University of Gothenburg, Gothenburg, Sweden; 3 Department of Internal Medicine and Clinical Nutrition, Institute of Biomedicine, Sahlgrenska Academy, University of Gothenburg, Gothenburg, Sweden; 4 Department of Internal Medicine, Kungälv Hospital, Kungälv, Sweden; 5 Department of Biology and Biological Engineering, Chalmers University of Technology, Gothenburg, Sweden; 6 Department of Internal Medicine, Södra Älvsborg Hospital, Borås, Sweden

**Keywords:** Ulcerative Colitis, Macrophages, TLR Signaling, TNF, Metabolome, CD, cluster of differentiation, CXCL, C-X-C motif chemokine ligand, ELISA, enzyme-linked immunosorbent assay, FLA, flagella, FS, fecal supernatant, GC-MS/MS, gas chromatography coupled to a tandem mass spectrometer, GM-CSF, granulocyte-macrophage colony-stimulating factor, IBD, inflammatory bowel disease, IFNγ, interferon γ, IL, interleukin, LP, lamina propria, LPS, lipopolysaccharide, M1MQ, M1 (proinflammatory) macrophage, ODN, oligodeoxynucleotides, PCA, principal component analysis, PGN, peptidoglycan, SCFA, short-chain fatty acid, Th, T-helper, TLR, Toll-like receptor, TNF, tumor necrosis factor, UC, ulcerative colitis

## Abstract

**Background & Aims:**

Intestinal macrophages adopt a hyporesponsive phenotype through education by local signals. Lack of proper macrophage maturation in patients with ulcerative colitis (UC) in remission may initiate gut inflammation. The aim, therefore, was to determine the effects of fecal luminal factors derived from healthy donors and UC patients in remission on macrophage phenotype and function.

**Methods:**

Fecal supernatants (FS) were extracted from fecal samples of healthy subjects and UC patients in remission. Monocytes were matured into macrophages in the presence of granulocyte-macrophage colony-stimulating factor without/with FS, stimulated with lipopolysaccharide, and macrophage phenotype and function were assessed. Fecal metabolomic profiles were analyzed by gas-chromatography/mass-spectrometry.

**Results:**

Fecal luminal factors derived from healthy donors were effective in down-regulating Toll-like receptor signaling, cytokine signaling, and antigen presentation in macrophages. Fecal luminal factors derived from UC patients in remission were less potent in inducing lipopolysaccharide hyporesponsiveness and modulating expression of genes involved in macrophage cytokine and Toll-like receptor signaling pathways. Although phagocytic and bactericidal abilities of macrophages were not affected by FS treatment, healthy FS-treated macrophages showed a greater ability to suppress cluster of differentiation 4^+^ T-cell activation and interferon γ secretion compared with UC remission FS-treated counterparts. Furthermore, metabolomic analysis showed differential fecal metabolite composition for healthy donors and UC patients in remission.

**Conclusions:**

Our data indicate that UC patients in remission lack luminal signals able to condition macrophages toward a hyporesponsive and tolerogenic phenotype, which may contribute to their persistent vulnerability to relapse.

## Graphical abstract

**Figure:**
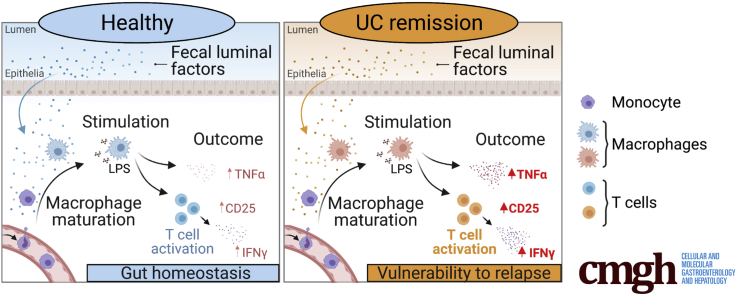

**Summary**

Fecal luminal factors from ulcerative colitis patients in remission, unlike from healthy individuals, fail to induce macrophage hyporesponsiveness, a feature typical of intestinal macrophages. This suggests a role for fecal luminal factors in guiding macrophage maturation under physiological conditions and disease.

Ulcerative colitis (UC) is an inflammatory bowel disease (IBD) characterized by chronic and relapsing inflammation that typically affects the rectum and colon. Complex interactions of multiple genetic and nongenetic determinants predispose individuals to IBD and eventually might culminate in aberrant and excessive immune responses against the intestinal microbiota.^1^

Macrophages of the lamina propria (LP) play a key role in sustaining the homeostasis of the intestine and are incessantly challenged by food antigens and commensal or (potentially) harmful bacteria. Intestinal macrophages have a hyporesponsive and tolerogenic nature^2^ and constantly are replenished from circulating monocytes.^3^^,^^4^ Once in the mucosa, local signals drive differentiation from inflammatory monocytes into hyporesponsive tissue resident macrophages.^2^^,^^5^ Down-regulation of cluster of differentiation (CD)14, receptors for IgA (CD89) and IgG (CD64, CD32, CD16) and the costimulatory molecules CD40, CD80, and CD86 is apparent after LP macrophage differentiation.^6^^,^^7^ Although intestinal macrophages express Toll-like receptors (TLRs), hyporesponsiveness to TLR ligands with decreased production of proinflammatory cytokines, such as tumor necrosis factor α (TNFα) and interleukin (IL)1β, is attributed to differential expression of downstream proteins involved in the TLR signaling pathways (eg, MYD88).^8^

Numerous data are present concerning macrophages during active inflammation in UC (reviewed by Caer and Wick^9^), but data regarding LP macrophages in UC during remission are rather limited. Although frequencies of LP macrophages are similar between UC remission and health, increased CD14 and TLR4 expression have been detected on colonic macrophages during remission.^10^ In addition, recent gene expression studies have shown the presence of a continuous inflammatory-like state and a gene dysregulation in colonic biopsy specimens from patients with quiescent UC.^11^, ^12^, ^13^

The immunomodulating determinants involved in the education of intestinal macrophages still are not fully defined, but intestinal microbiota and microbiota-derived metabolites increasingly are recognized for their role in imprinting tissue-specific features of intestinal macrophages.^5^^,^^14^ In addition to this, it is known that UC patients show a dysbiotic microbiota composition, including a decrease in *Firmicutes* and *Bacteroidetes* phyla^15^^,^^16^ and altered fecal metabolite profiles,^17^ which could affect intestinal macrophage maturation. Thus, the microbiota-induced hyporesponsive state of the intestinal LP macrophages may be lost in UC patients in remission, eventually leading to relapse.

We hypothesize that fecal luminal factors polarize intestinal macrophages toward hyporesponsiveness during health, and that this polarization process is impaired in UC patients. Therefore, the present study aimed to determine the effects of fecal luminal factors on macrophage phenotype and function and to compare the macrophage polarizing effects of fecal luminal factors derived from healthy donors and UC patients in remission. In addition, we examined the fecal metabolite composition from healthy donors and UC patients.

## Results

### Fecal Luminal Factors From Healthy Donors Promote Lipopolysaccharide Hyporesponsiveness in Macrophages

**Figure 1:**
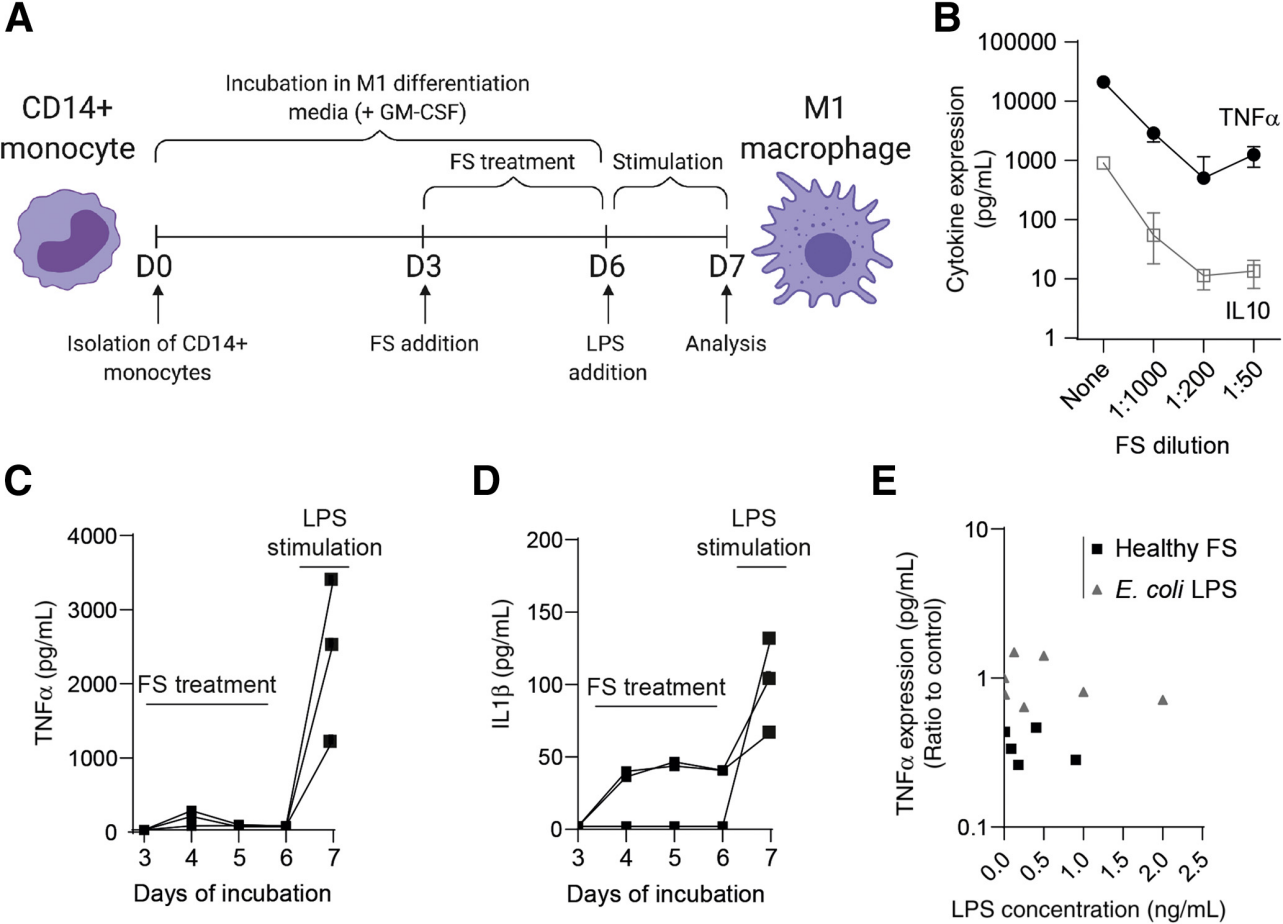
**Fecal luminal factors from healthy subjects reduce macrophage responsiveness to LPS.** (*A*) The model of experimental set-up. Isolated CD14^+^ monocytes from healthy blood donors were matured into M1 macrophages (M1MQs) in a GM-CSF–containing media for 6 days. On days 3–6 of maturation, FS were added. On day 6, cells were washed and the M1MQs were stimulated with LPS (10 ng/mL) for 24 hours. TNFα and IL10 were measured in the supernatants using ELISA. (*B*) TNFα and IL10 expression on day 7 from M1MQs treated without FS or with FS from healthy subjects diluted 1:1000, 1:200, and 1:50. Values indicate median (interquartile range), n = 10. (*C*) TNFα and (*D*) IL1β expression during FS treatment (days 3–6) and after LPS stimulation (day 7) using FS from healthy subjects, diluted 1:1000 (n = 3). (*E*) Correlation between TNFα expression in the supernatants on day 7 and LPS concentration in the media during days 3–6. The concentration of LPS was determined in 5 FS samples. Next, cells were conditioned from days 3 to 6 with the 5 FS samples, diluted 1:1000, or *E coli* LPS within the same concentration range as the FS samples. The M1MQs then were stimulated with LPS for 24 hours and TNFα expression was measured on day 7. Pearson correlation analyses showed no significant correlation (r = -0.04; *P* = .90). All analyses were performed in triplicate (shown as means).

To investigate the effects of fecal luminal factors on macrophages, CD14^+^ monocytes were matured into proinflammatory M1 macrophages (M1MQs) for 6 days with granulocyte-macrophage colony-stimulating factor (GM-CSF), without or with fecal supernatants (FS) from healthy subjects during days 3–6 and subsequently stimulated with lipopolysaccharide (LPS) (schematic overview in Figure 1*A*). Overall, TNFα and IL10 expression from the M1MQs decreased with increasing concentrations of FS, reaching a steady-state at dilutions 1:200 to 1:50 (Figure 1*B*). Based on its location in the dynamic phase, the 1:1000 FS dilution was chosen and used throughout the study. To rule out the possibility of the FS samples inducing stimulation of the M1MQs between days 4–6 and thereby exhausting the cells, TNFα and IL1β expression was monitored during the FS treatment. Results showed negligible expression of TNFα on days 4–6, while a clear peak was detected only after LPS stimulation (Figure 1*C*). For IL1β, 2 of 3 FS samples induced expression on days 4–6, followed by an increase after LPS stimulation (Figure 1*D*), however, levels were consistently low throughout induction and IL1β expression on day 7 was similar/slightly lower than levels detected from FS-untreated macrophages (median, 146 pg/mL). We also examined whether conditioning with LPS alone could generate similar results and M1MQs were conditioned in parallel with FS samples and *Escherichia coli* LPS within the same concentration. There was no correlation between TNFα expression and LPS conditioning concentration in any of the groups (Figure 1*E*).

### Fecal Luminal Factors From UC Patients Have Impaired Potency to Condition Macrophages

**Figure 2:**
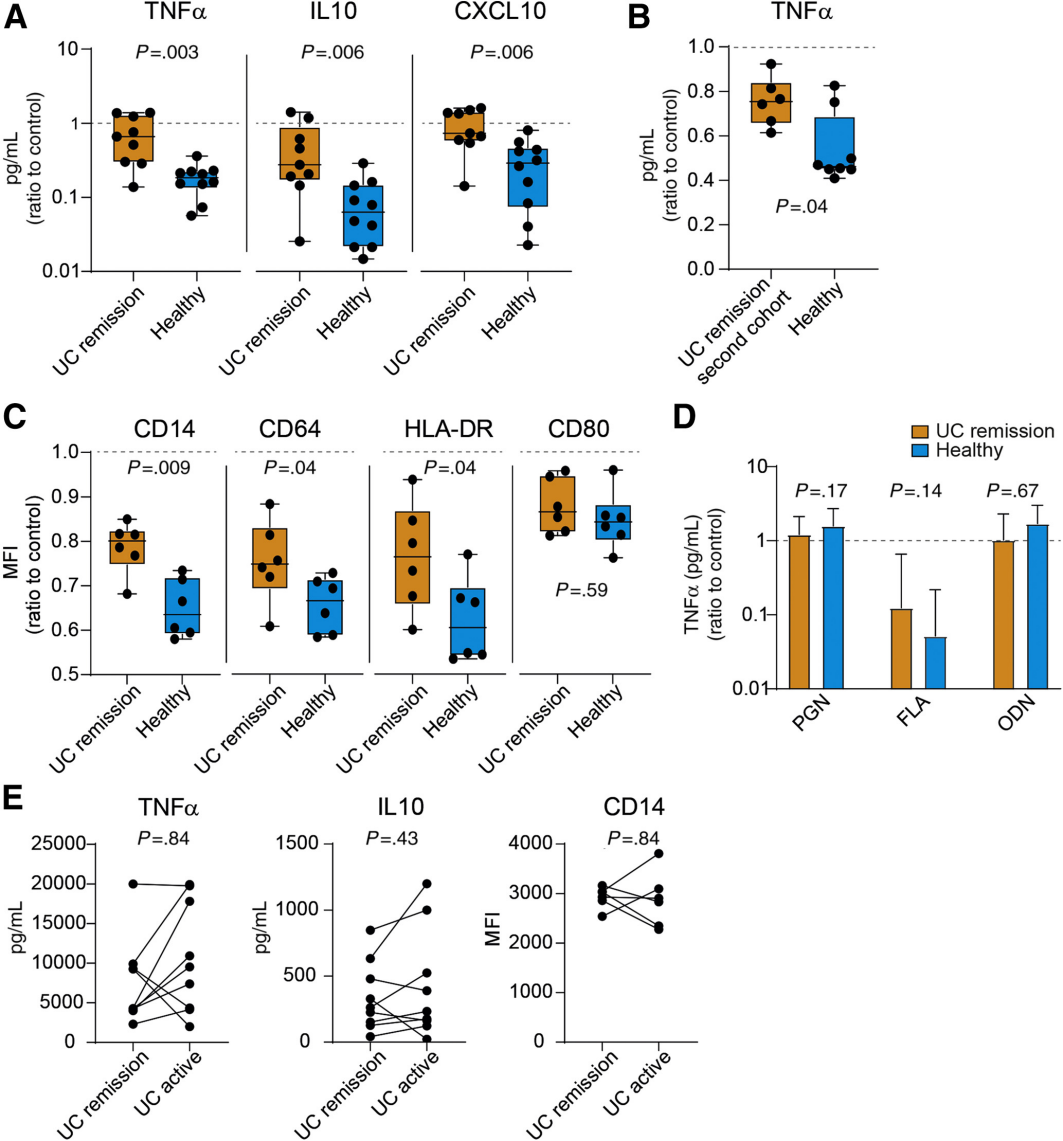
**Macrophages treated with FS from healthy donors are less responsive to LPS than cells treated with FS from UC patients in remission.** CD14^+^ monocytes were matured into M1 macrophages in a media containing GM-CSF for 6 days, without or with FS diluted 1:1000 during days 3–6. On day 6, the M1 matured cells were stimulated with (*A–C* and *E*) 10 ng/mL LPS or (*D*) 10 μg/mL PGN, 50 ng/mL flagella (FLA), and 5 μmol/L oligodeoxynucleotides (ODN). Cytokine production was measured by ELISA and cell surface marker median fluorescent intensity (MFI) was measured by by flow cytometry. (*A*) LPS-induced TNFα, IL10, and CXCL10 expression from macrophages treated with FS from UC patients in remission (n = 9) and healthy subjects (n = 10). (*B*) TNFα production in response to LPS in macrophages treated with FS from the second cohort of UC patients in remission (n = 6) and healthy subjects (n = 8). (*C*) Expression of surface markers on macrophages treated with UC remission FS (n = 6) and healthy subject FS (n = 6). The macrophages were gated as CD64^+^HLA-DR^+^7-amino actinomycin D^-^ cells and MFI was defined for CD14, CD64, HLA-DR, and CD80. (*D*) TNFα production in response to PGN, FLA, and ODN. (*D*) Bars indicate median values (interquartile range). (*E*) LPS-induced TNFα and IL10 expression in the supernatants and surface expression of CD14 from macrophages treated with FS from UC patients in remission and the same individuals during active disease (n = 9 for TNFα and IL10 and n = 6 for CD14). The macrophages were gated as in panel *C*. The same individuals are connected by *lines*. (*A–D*) Data are shown as the ratio of FS treated over FS untreated (control). (*A–D*) Significance was assessed by the Mann–Whitney *U* test and (*E*) by the Wilcoxon test. *Dotted lines* indicate control (ratio = 1). All analyses were performed in triplicate (shown as means).

Using the set-up in Figure 1*A*, M1MQs treated with FS from UC patients in remission showed higher TNFα, IL10, and C-X-C motif chemokine ligand 10 (CXCL10) expression in response to LPS stimulation as compared with healthy FS (Figure 2*A*). Expression did not differ between UC remission FS-treated cells and control cells, shown by overlap of the UC remission group and the dotted line for control (Figure 2*A*). The TNFα response of M1MQs treated with FS from UC patients in remission and healthy subjects also was confirmed in a separate cohort of UC patients in remission (Figure 2*B*). Next, the M1MQs were analyzed for surface marker expression and healthy FS induced a larger reduction of CD14, CD64, and HLA-DR expression as compared with UC remission FS, whereas no difference was observed in CD80 expression between the 2 groups (Figure 2*C*). To compare the response of UC remission and healthy FS-treated M1MQs with other TLR ligands, cells were pretreated with FS and stimulated with flagella (FLA) (TLR5), peptidoglycan (PGN) (TLR2), and oligodeoxynucleotides (ODN) (TLR9) on day 6 and TNFα expression was examined. Cells treated with UC remission and healthy FS responded similarly to PGN, FLA, and ODN (Figure 2*D*). For FLA only, the addition of FS induced hyporesponsiveness as compared with control, shown by no overlap with the dotted control line (Figure 2*D*).

Although the focus of this study was to compare the effects of luminal factors from UC patients in remission and healthy subjects, cells also were treated with FS from the UC patients during active disease. UC active and UC remission FS-treated cells did not differ in their TNFα and IL10 production or their surface expression of CD14 (Figure 2*E*).

### Fecal Luminal Factors From UC Patients in Remission Have Impaired Potency to Regulate Macrophage Gene Expression and Pathway Scores Toward Hyporesponsiveness

**Figure 3:**
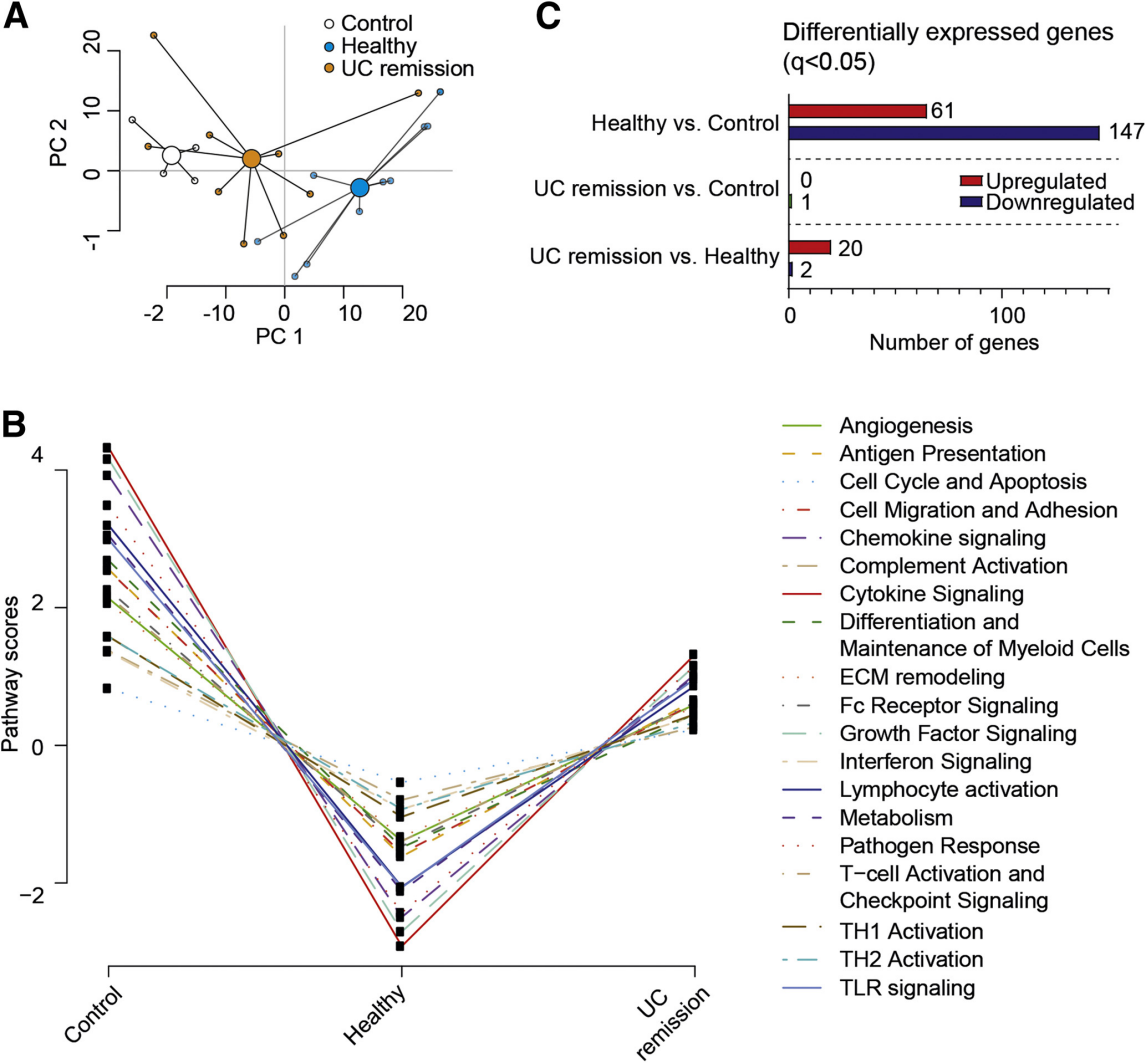
**Macrophage gene expression and pathway scores are altered by fecal luminal factors.** CD14^+^ monocytes were matured into M1 macrophages in GM-CSF–containing media for 6 days without (control, n = 4) or with FS diluted 1:1000 from UC patients in remission (n = 9) and healthy subjects (n = 10). On day 6, the M1MQs were stimulated with LPS (10 ng/mL) for 24 hours. Expression of 738 genes was measured using the NanoString nCounter Myeloid-Innate-Immunity Panel. (*A*) Principal component analysis for control, UC remission FS, and healthy FS. (*B*) NanoString Advanced Analysis graphic display of pathway scores. *Lines* show each pathway’s average score for the 3 groups. (*C*) Pairwise analysis of differentially expressed genes (q < 0.05). The number of up-regulated or down-regulated genes are shown to the right of the *bars*. Cells from 6 separate wells from each individual were pooled and used for analysis. ECM, extracellular matrix; Fc, Fragment, crystallizable.

**Figure 4:**
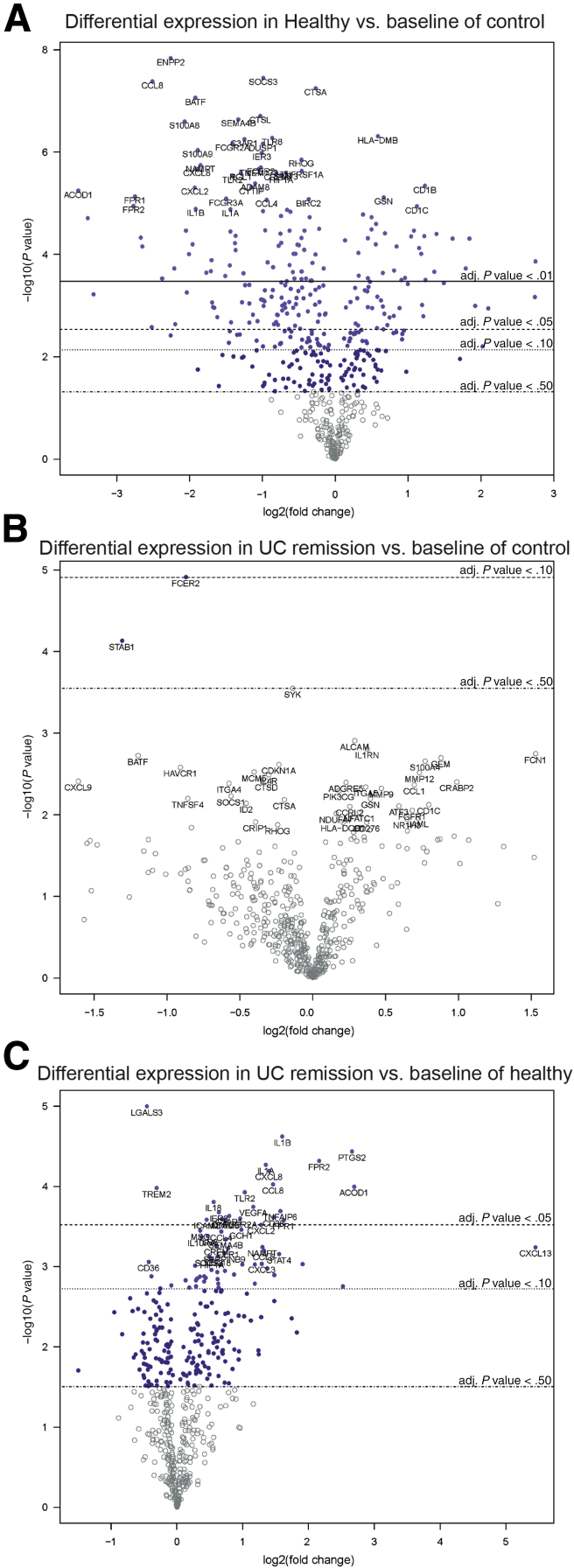
**Pairwise differential gene expression between control, healthy FS, and UC remission FS–treated macrophages.** CD14^+^ monocytes were matured into M1 macrophages in a media-containing GM-CSF for 6 days, without or with FS diluted 1:1000 during days 3–6. On day 6, the M1 matured cells were stimulated with LPS for 24 hours followed by gene expression analysis using the NanoString nCounter Myeloid-Innate-Immunity Panel. (*A*) Healthy FS-treated cells vs control, (*B*) UC remission FS–treated cells vs control, and (*C*) UC remission FS vs healthy FS-treated cells. Differential gene expression of 738 genes are shown in the volcano plots showing log_2_ fold change of the gene expression vs significance (Student *t* test). False-discovery rate analysis was performed using the Benjamini–Yekutieli method. Control, n = 4; healthy, n = 10; and UC remission, n = 9. adj, adjusted.

To examine the transcriptional profiles of FS-treated and untreated macrophages after LPS stimulation, the NanoString nCounter Myeloid-Innate-Immunity Panel (NanoString Technologies, Inc, Seattle, WA) was used. A principal component analysis (PCA) based on 738 genes showed a large separation between control and healthy FS-treated macrophages, with UC remission FS-treated cells placed in between (Figure 3*A*). Using NanoString pathway analysis, the mean scores for 19 different pathways showed clear down-regulation for healthy FS and intermediate down-regulation for UC remission FS (Figure 3B). In detail, healthy FS induced lower pathway scores for all 19 pathways in relation to control, UC remission showed no change from control, and 13 pathway scores differed between UC remission and healthy subjects (Table 1). Pairwise comparisons showed major changes in gene expression between healthy and control cells, negligible changes between UC remission and control cells , and some alterations between UC remission and healthy cells (Figures 3C and 4).

**Table 1:** NanoString Advanced Analysis Pathway Score Comparisons Between Control (Untreated, n = 4), Healthy FS-Treated (n = 10), and UC Remission FS–Treated (n = 9) Macrophages

Pathway	Control	Healthy subjects	UC remission	*P* value^a^ control vs healthy	*P* value control vs UC	*P* value healthy vs UC
Angiogenesis	2.04 (1.68–2.79)^b^	-1.53 (-3.49 to 1.05)	1.22 (-3.25 to 2.36)	*.003*	.38	.07
Antigen presentation	2.44 (2.13–3.22)	-1.94 (-3.25 to 0.37)	1.02 (-2.85 to 2.99)	*.003*	.49	*.04*
Cell cycle and apoptosis	0.82 (0.52–1.16)	-0.64 (-1.22 to 0.24)	0.31 (-0.96 to 1.01)	*.005*	.61	.06
Cell migration and adhesion	2.43 (2.08–3.23)	-1.94 (-3.17 to 0.72)	0.93 (-2.76 to 2.99)	*.002*	.42	*<.05*
Chemokine signaling	3.69 (3.21–5.09)	-2.78 (-4.46 to 0.55)	1.16 (-3.60 to 4.31)	*.002*	.46	*.03*
Complement activation	1.31 (1.06–1.81)	-0.79 (-1.79 to 0.12)	0.11 (-1.63 to 1.60)	*.003*	.36	.08
Cytokine signaling	4.13 (3.33–5.71)	-3.16 (-5.65 to 0.95)	1.56 (-4.95 to 5.10)	*.003*	.49	*.04*
Differentiation and maintenance of myeloid cells	2.51 (2.02–3.70)	-1.71 (-2.01 to -0.25)	-0.28 (-1.38 to 3.39)	*.002*	.49	*.03*
Extracellular matrix remodeling	1.91 (1.53–2.91)	-1.60 (-2.78 to 0.38)	0.79 (-2.29 to 2.48)	*.003*	.49	*.04*
Fc receptor signaling	2.15 (1.81–2.93)	-1.63 (-3.44 to 0.95)	1.06 (-3.12 to 2.52)	*.003*	.49	*.04*
Growth factor signaling	3.88 (3.32–5.55)	-3.05 (-5.70 to 1.16)	1.60 (-5.20 to 4.67)	*.003*	.49	*.04*
IFN signaling	1.46 (0.59–1.93)	-1.21 (-2.09 to 0.49)	0.79 (-2.35 to 1.95)	*.01*	.63	.12
Lymphocyte activation	3.09 (2.61–4.00)	-2.34 (-4.82 to 1.25)	1.34 (-3.98 to 3.57)	*.002*	.42	*<.05*
Metabolism	2.94 (2.17–4.14)	-2.36 (-4.62 to 0.77)	1.26 (-3.76 to 3.87)	*.004*	.68	*.04*
Pathogen response	3.22 (2.74–4.77)	-2.75 (-3.96 to 0.05)	0.82 (-3.02 to 4.18)	*.004*	.74	*.02*
T-cell activation and checkpoint signaling	2.08 (1.70–2.82)	-1.88 (-2.96 to 0.66)	0.91 (-2.46 to 2.44)	*.002*	.40	.06
TH1 activation	1.52 (1.45–2.14)	-1.25 (-1.86 to 0.29)	0.44 (-1.56 to 1.84)	*.002*	.54	*.03*
TH2 activation	1.52 (1.14–2.12)	-1.15 (-1.76 to 0.50)	0.31 (-1.60 to 1.88)	*.002*	.31	.07
TLR signaling	2.88 (2.30–3.91)	-2.36 (-3.84 to 0.45)	1.43 (-3.07 to 3.51)	*.002*	.44	*.04*

NOTE. The expression of 738 genes was measured using the NanoString nCounter Myeloid-Innate-Immunity Panel.

Fc, Fragment, crystallizable.

a Significance was determined using the Kruskal–Wallis test followed by the Dunn multiple comparisons test. Significant differences are indicated in italics.

b Data show pathway scores as the median (range).

### UC Remission FS-Treated Macrophages Elicit a Distinct Expression of TLR Signaling–Associated Genes on LPS Stimulation

**Figure 5:**
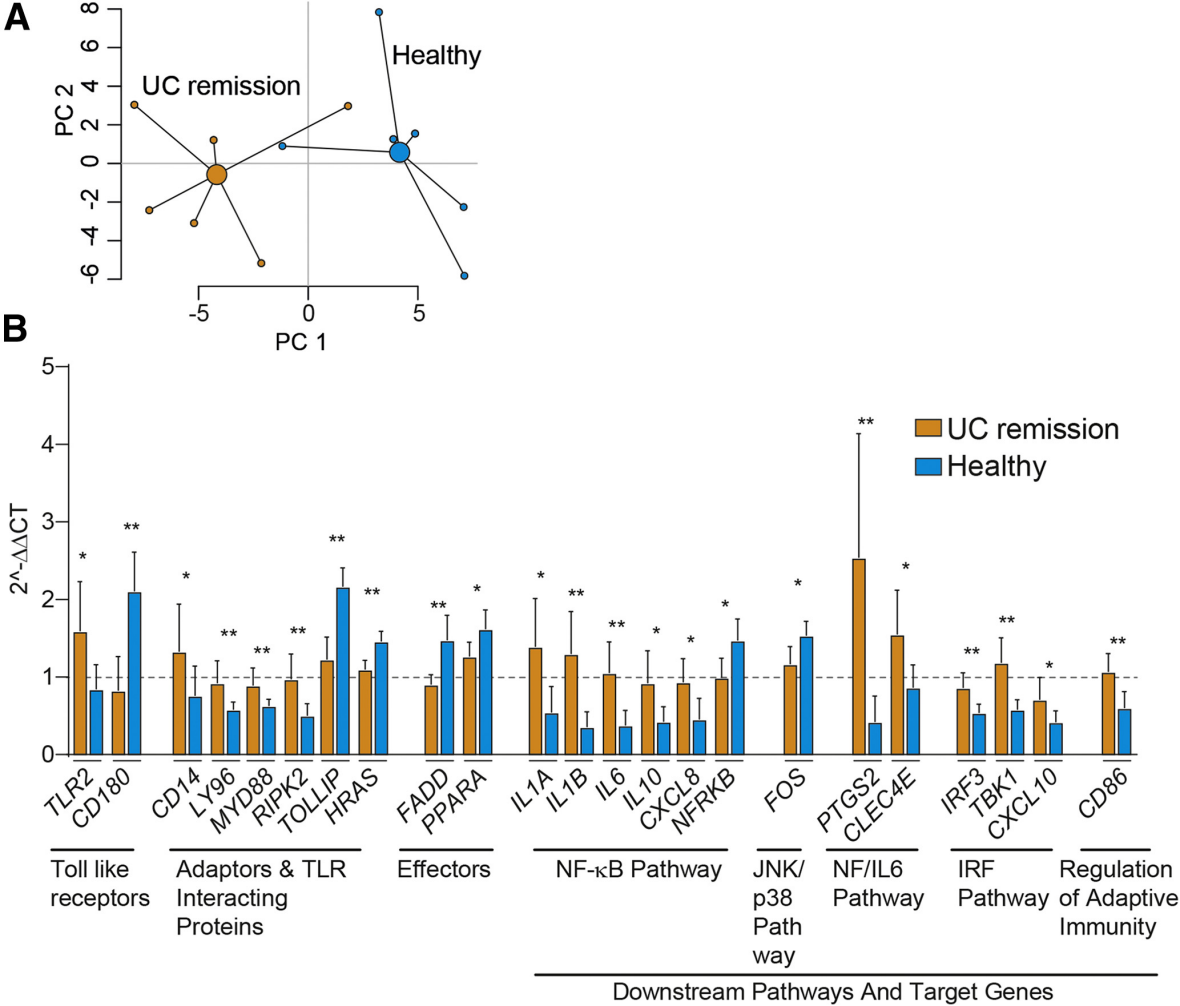
**TLR signaling–associated genes are differentially expressed in UC remission vs healthy FS-treated macrophages after LPS stimulation.** CD14^+^ monocytes were matured into M1 macrophages in GM-CSF–containing media for 6 days without (control, n = 3) or with FS diluted 1:1000 from UC patients in remission (n = 6) and healthy subjects (n = 6). On day 6, the M1MQs were stimulated with LPS (10 ng/mL) for 24 hours. A TLR pathway–focused rtPCR array was used to quantify gene expression of the macrophages. (*A*) Principal component analysis based on expression of the 81 expressed genes for UC remission FS and healthy FS calculated by the 2^−ΔCT^ method. (*B*) Comparison of gene expression levels between UC FS-treated cells vs healthy FS-treated cells. Fold change was calculated by comparing gene expression of the FS-treated cells with controls by the 2^−ΔΔCT^ method. *Bars* indicate median values (interquartile range). Mann–Whitney *U* test, ∗*P* < .05, ∗∗*P* < .01. The *dotted line* indicates control (ratio = 1). Cells from 6 separate wells from each individual were pooled and used for analysis. IRF, interferon regulatory factor; JNK, c-Jun N-terminal kinase; NF, nuclear factor; NF-κB, nuclear factor-κB.

To further evaluate the role of TLR signaling we examined gene expression using a TLR-focused polymerase chain reaction (reverse transcription (rt) PCR) array. A global overview of gene expression for UC remission and healthy FS-treated macrophages in a PCA showed clustering of the data (Figure 5*A*). Furthermore, 23 of 81 genes analyzed showed differential expression between healthy and UC remission FS-treated M1MQs (Figure 5*B*). Genes involved receptors, members of signaling complexes, effectors, members of downstream pathways (nuclear factor-κB, c-Jun N-terminal kinase (JNK)/p38, nuclear factor (NF)/IL6, and interferon regulatory factor (IRF) pathways), and regulators of adaptive immunity. A majority of the genes showed higher expression in UC remission FS-treated cells while only *CD180*, *TOLLIP*, *HRAS*, *FADD*, *PPARA*, *NFRKB*, and *FOS* were higher when treated with healthy FS (Figure 5*B*). However, no differences were detected for TLR4. Taken together, our results indicate that fecal luminal factors from healthy subjects strongly alters TLR signaling in macrophages and renders the cells less responsive to LPS while only minor effects are imprinted by the fecal luminal factors from UC patients in remission.

### The Phagocytic and Bactericidal Abilities of Macrophages Are Unaffected by Fecal Luminal Factors

**Figure 6:**
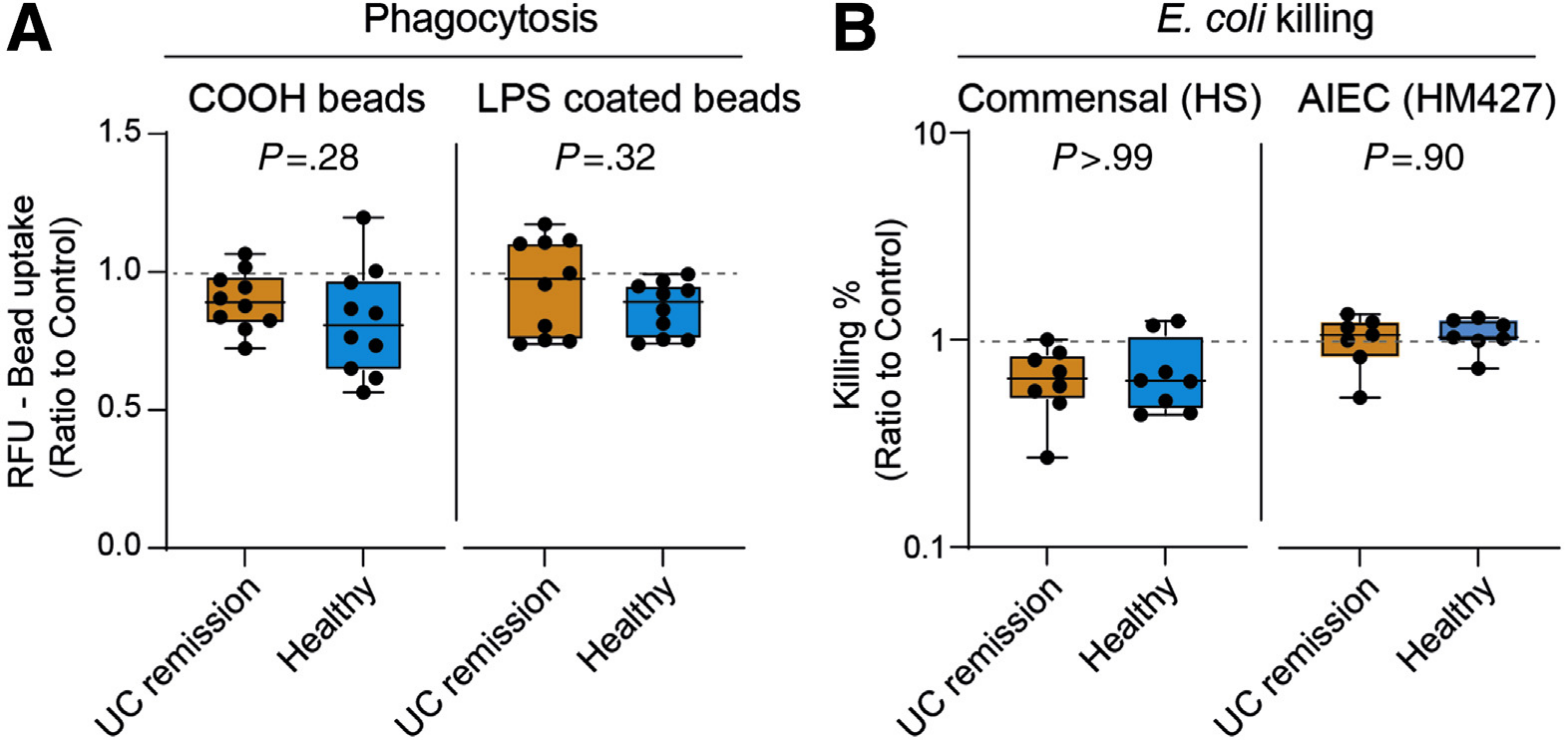
**UC and healthy FS-treated macrophages display similar phagocytic and bactericidal abilities.** CD14^+^ monocytes were matured into M1 macrophages in GM-CSF–containing media for 6 days without or with FS diluted 1:1000 from UC patients in remission and healthy subjects. (*A*) Matured and FS-treated macrophages were stimulated with LPS for 24 hours and incubated with fluorescent carboxylated (COOH) or LPS-coated beads for 30 minutes. Phagocytosis of beads was measured using a microplate reader. UC remission (n = 10), healthy subjects (n = 10). (*B*) Bacterial killing of *E coli*. Matured and FS-treated macrophages were infected with the commensal *E coli* HS or adherent-invasive *E coli* (AIEC) HM427, bacterial survival within macrophages was quantified at 15 minutes (T15) and at 120 minutes (T120) after infection, respectively, using the gentamycin protection assay. The bacterial killing percentage was calculated as follows: ([T15-T120]/T15) ∗ 100. *E coli* HM: UC remission (n = 8), healthy subjects (n = 8); *E coli* HS427: UC remission (n = 7), healthy subjects (n = 7). Significance was assessed by the Mann–Whitney *U* test. *Dotted lines* indicate control (ratio = 1). All analyses were performed in triplicate or quadruplicate (shown as means). RFU, relative fluorescence unit.

To investigate whether FS treatment can induce functional changes in macrophages, the cells were analyzed for their phagocytic and bacterial killing ability after FS treatment. To mimic apoptotic cell and bacterial uptake, macrophages were co-incubated with carboxylated and LPS-coated beads, respectively. Both carboxylated and LPS-coated beads were phagocytosed at similar levels by macrophages treated with UC remission and healthy FS (Figure 6*A*). Bactericidal activity toward the commensal *E coli* strain HS and the adherent-invasive *E coli* strain HM427 also were similar between the 2 groups (Figure 6*B*).

### UC Remission FS-Treated Macrophages Have an Impaired Capacity to Suppress CD4^+^ T-Cell Activation and T-Cell Cytokine Secretion

**Figure 7:**
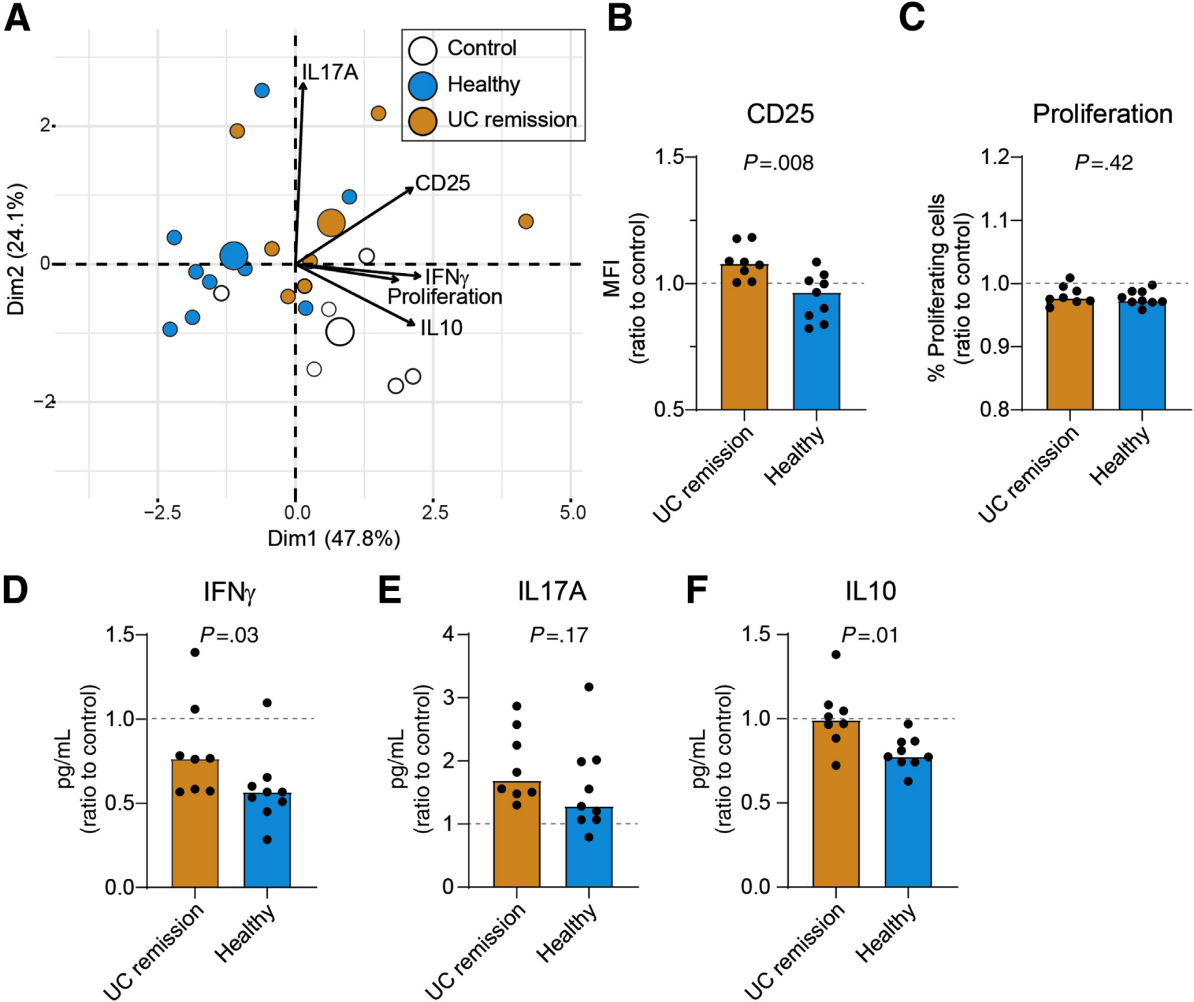
**Macrophages treated with FS from healthy subjects reduced CD4**^**+**^**T-cell activation and cytokine secretion as compared with UC remission and control.** CD14^+^ monocytes were matured into M1 macrophages in GM-CSF–containing media for 6 days without (control) or with FS diluted 1:1000 from UC patients in remission and healthy subjects during days 3–6 and stimulated with LPS overnight. Purified autologous CD4^+^ T cells were added to the macrophages (ratio 1:1) with the presence of anti-CD3 for 3 days. T-cell proliferation (carboxyfluorescein diacetate succinimidyl ester) and activation (CD25) were analyzed by flow cytometry and cytokine expression in the supernatants by ELISA. (*A*) Principal component analysis biplot of CD25 expression, proliferation, and cytokine expression (IL17A, IFNγ, IL10) for UC remission, healthy subjects, and controls. *Large dots* indicate the weighted means of the groups (centroids). *Arrows* point toward increased expression. (*B*) Median fluorescent intensity (MFI) of CD25 on CD4^+^ T cells, (C) the percentage of proliferating T cells defined by carboxyfluorescein diacetate succinimidyl ester, and secretion of (*D*) IFNγ, (*E*) IL10, and (*F*) IL17A into the supernatants are shown as the ratio of FS treated over control. *Bars* indicate median values. Significance was assessed by the Mann–Whitney *U* test. UC remission, n = 8; healthy subjects, n = 9; and controls, n = 6. *Dotted lines* indicate controls (ratio = 1). All analyses were performed in triplicate (shown as means).

To further examine the functional properties of FS-treated cells, macrophages were evaluated for their ability to activate CD4^+^ T cells in an autologous mixed lymphocyte reaction. CD4^+^ T cells were added to control (untreated), UC remission, or healthy FS-treated M1MQs after LPS stimulation. Effects on T cells were evaluated after 3 days of studying CD25 surface expression, proliferation, and cytokine production profiles (interferon γ [IFNγ], IL10, IL17A). A PCA biplot for the autologous mixed lymphocyte reaction visualized that healthy FS-treated macrophages induced lower levels of CD25 on T cells, reduced proliferation, and decreased IFNγ and IL10 production compared with UC remission FS-treated and control macrophages (Figure 7*A*). In contrast, healthy and UC remission FS-treated macrophages had a similar effect on IL17 production by T cells and increased the levels as compared with control (Figure 7*A*). Hotelling’s T2 tests showed significant between-group differences based on the 5 variables analyzed: UC remission vs healthy (*P* = .002), healthy vs control (*P* = .0005), and UC remission vs control (*P* = .0009).

Univariate comparisons for the autologous mixed lymphocyte reaction parameters between UC remission and healthy FS-treated macrophages showed lower CD25 surface expression and IFNγ and IL10 expression for healthy compared with UC remission, whereas no differences were detected for proliferation or IL17A (Figure 7*B–F*).

### UC Remission and Healthy FS Display Different Metabolomic Profiles

**Figure 8:**
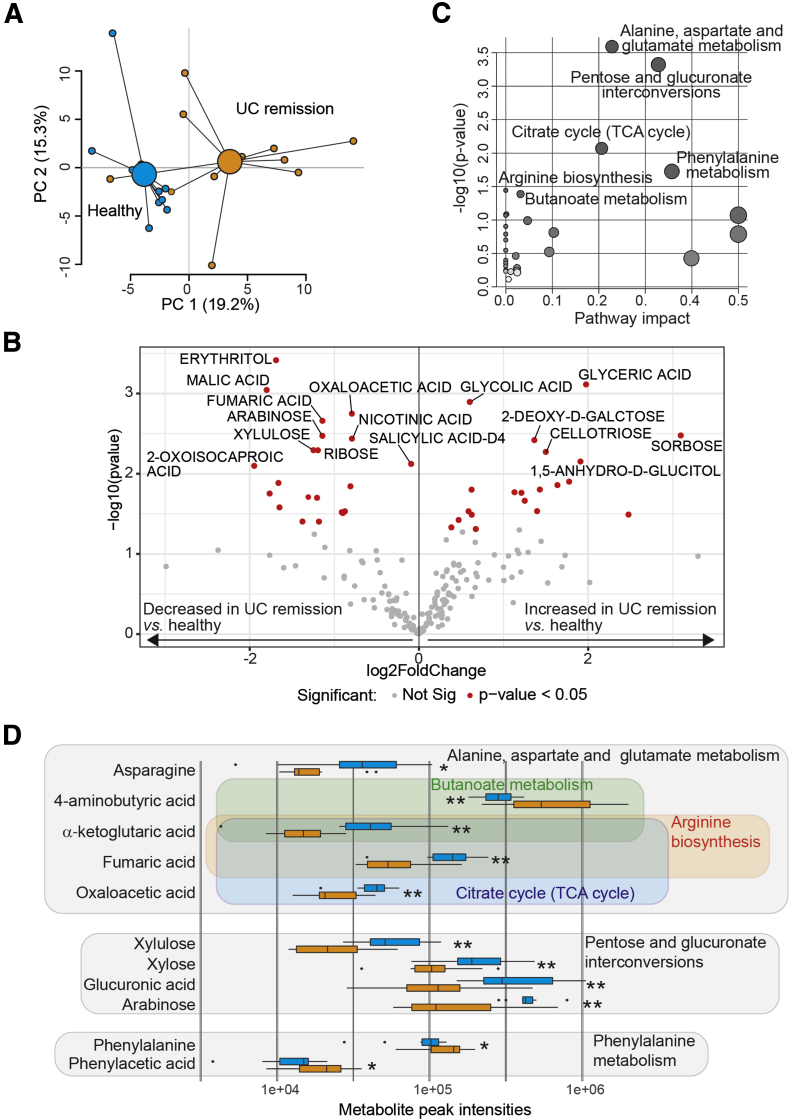
**The fecal metabolomic profiles differ between UC patients in remission and healthy subjects.** Metabolomic analysis was performed in fecal supernatants using GC-MS/MS for healthy subjects (n = 10) and UC patients in remission (n = 11). Pathway analysis with the significantly different metabolites (n = 41) was performed using MetaboAnalyst. (*A*) Principal component analysis and (*B*) volcano plot showing fecal metabolite abundance levels vs significance based on 201 fecal metabolites. Significantly altered metabolites in panel *B* are indicated in red (*P* < .05) and metabolites with a *P* value less than .01 are annotated. Significance was assessed by the Student *t* test. (*C*) MetaboAnalyst pathway analysis of differential metabolites. The size of the circles indicates pathway impact, the higher pathway impact the larger circle. The color of the circles indicates *P* value, the darker circle the lower *P* value. Significant pathways are annotated. (*D*) Depiction of metabolites linked to the significant pathways identified in panel *C*. Metabolites involved are encircled for each pathway. Metabolite peak intensities are shown for healthy subjects (blue) and UC patients in remission (orange). Significance was assessed by the Mann–Whitney *U* test, ∗*P* < .05, ∗∗*P* < .01. Not Sig, not significant; TCA, tricarboxylic acid.

To explore if fecal metabolite profiles differed between UC patients in remission and healthy subjects, a metabolomic analysis was performed. A PCA based on 201 metabolites showed a clear separation between UC remission and healthy FS (Figure 8*A*). Among the metabolites, 20 were more abundant in UC remission while 21 of the metabolites were represented more among healthy subjects (Figure 8*B*). To investigate the biological pathways involved in the metabolic signatures, pathway analysis was conducted using the significantly altered metabolites. Alanine, aspartate, and glutamate metabolism; pentose and glucuronate interconversions; citrate cycle (tricarboxylic acid (TCA) cycle); phenylalanine metabolism; arginine biosynthesis; and butanoate metabolism were identified as significant pathways (Figure 8*C*). Matched metabolites involved in these pathways identified α-ketoglutaric acid and fumaric acid in 3 of 6 pathways (Figure 8*D*). The ratio between α-ketoglutaric acid and succinic acid, promoting classically activated macrophages,^18^ was calculated and found to be lower for UC patients in remission than for healthy subjects (0.36 [0.21–0.82] vs 1.12 [0.20–3.31]; *P* = .003, values indicate median [range]).

Last, we analyzed short-chain fatty acids (SCFAs) in the FS samples, but no differences could be detected between healthy and UC patients in remission or between UC patients in remission and UC patients with active disease (Table 2).

**Table 2:** Peak Intensities of Short-Chain Fatty Acids in Fecal Supernatants From Healthy Subjects, UC Patients in Remission, and UC Patients During Active Disease

	Healthy subjects	UC remission	*P* value^a^	UC active	*P* value^b^
Acetic acid	589 (318–1407)^c^	694 (168–1120)	.47	662 (283–940)	.28
Proprinoic acid	104 (45–278)	161 (66–457)	.13	114 (44–259)	.27
Butyric acid	78 (25–159)	72 (23–678)	.77	84 (16–198)	.76
Isobutyric acid	7.9 (0.6–17.5)	11.7 (0.7–41)	.35	11.7 (3.4–38.2)	.64
Isovaleric acid	3.8 (0.2–12.7)	7.8 (1.5–33.4)	.29	5.3 (3.4–22.9)	.52
Valeric acid	5.7 (0.9–24.2)	6.7 (1.0–113.2)	.74	4.3 (0.5–17.9)	.20
Caproic acid	5.7 (0.1–59.1)	4.8 (0.3–14.8)	.85	1.2 (0.0–10.1)	.33

a Between healthy subjects and UC remission, Mann–Whitney *U* test.

b Between UC remission and UC active, Wilcoxon test.

c Data show peak intensity as the median (range).

## Discussion

The current study shows that fecal luminal factors modulate macrophage phenotype and function. It also shows that fecal luminal factors derived from UC patients in remission are insufficient in inducing LPS hyporesponsiveness and modulating genes involved in cytokine and TLR signaling pathways in macrophages. Moreover, macrophages treated with UC remission FS are deficient in suppressing activation and cytokine production of CD4+ T cells. Finally, UC patients in remission display an altered fecal metabolomic profile compared with healthy subjects.

The human gut is colonized by a vast number of gram-negative bacteria that constantly shed LPS. The LPS concentration in the colonic lumen is approximated to reach as high as 50 μg/mL.^19^^,^^20^ Given that LPS is a potent inducer of proinflammatory response in macrophages and the gut is the largest reservoir of macrophages, tight regulation of LPS response is crucial to maintain gut homeostasis. In fact, promoters associated with LPS response and/or monocyte-to-macrophage differentiation are enriched among identified IBD susceptibility loci.^21^ Previously, it has been shown that fecal bacteria derived from UC patients are more effective than fecal bacteria from healthy subjects in inducing proinflammatory surface markers and cytokine expression of blood monocytes.^22^ Here, we assessed the effects of fecal luminal factors that, in contrast to the microbiota itself, are more likely to come in contact with macrophages. Because intestinal macrophages are derived from blood monocytes, we used primary cells instead of cell lines to ensure biological relevance, and not adding FS until day 3 obtained equal initial polarizing effects of GM-CSF followed by a subsequent encounter of luminal factors. Our results of down-regulation of CD14 and greatly reduced cytokine response to LPS stimulation in macrophages treated with FS derived from healthy individuals but not from UC patients indicate the importance of proper macrophage education by the local environment for gut homeostasis and are in line with data reported from intestinal macrophages.^23^, ^24^, ^25^ Furthermore, the immune pathway scoring analysis indicated that the reaction potential among UC remission FS-treated macrophages was consistently higher as compared with healthy FS-treated macrophages.

Intestinal macrophages have a high threshold to activate the nuclear factor-κB pathway in response to LPS stimulation.^8^ However, this control of inflammatory pathways seems impaired in UC patients during remission because the TLR signaling pathway and numerous TLR-associated genes were up-regulated in UC remission FS-treated cells as compared with healthy FS-treated cells. On the other hand, *CD180* (RP105), a TLR4 homologue and inhibitor of LPS-induced cytokine production in macrophages,^26^ was expressed more in healthy FS-treated cells. The marked down-regulation of receptor-interacting serine/threonine-protein kinase 2 (*RIPK2*), involved in transmitting signals from various immune receptors the up-regulation of the inhibitory adaptor molecule Toll Interacting Protein (*TOLLIP*); and the regulators Fas-associated protein with death domain (*FADD*) and peroxisome proliferator-activated receptor alpha (*PPARA*) further suggest that the fecal luminal factors from healthy subjects were more effective than fecal luminal factors from UC patients in imprinting properties that would favor gut homeostasis.^27^, ^28^, ^29^, ^30^

Concerning function, neither the macrophages phagocytic ability nor bacterial killing ability of 2 different *E coli* strains were affected by FS treatment. In fact, despite the paucity in cytokine production, intestinal macrophages are as capable as blood monocytes in their phagocytosis and bacterial killing abilities, suggesting that these properties are unaffected by local signals in the intestine.^25^ Furthermore, impaired bactericidal activity is not a dysfunction associated with UC.^31^ On the other hand, commensal bacterial–derived local signals are crucial for proper macrophage/CD4^+^ T-cell interaction in lamina propria.^14^^,^^32^ During steady-state conditions, intestinal macrophages regulate effector T-cell responses and promote maintenance of regulatory T cells.^32^^,^^33^ However, in an IBD setting and/or in the absence of homeostatic signals, inflammatory macrophages induce pathogenic T helper (Th)1 and Th17 responses.^14^^,^^32^^,^^34^^,^^35^ Consistent with this, our autologous mixed lymphocyte reaction data showed that UC remission FS-treated M1MQs showed impaired suppression on activation and cytokine expression of CD4^+^ T cells, which fits with the antigen presentation, lymphocyte activation, and Th1 pathway analysis scores from the NanoString analysis. We also noted that FS induced expression of IL17A, a cytokine known both as proinflammatory and protective for the barrier surface, depending on the context. Mechanisms of T-regulatory cell generation from FS-treated macrophages still warrant further investigation.

Although the signals involved in monocyte-to-macrophage differentiation under homeostatic conditions have yet to be defined, there is compelling evidence that microbiota and microbiota-derived signals play a crucial role. Interestingly, antibiotic treatment induces hyper-responsive intestinal macrophages mediating long-term and pathologic T-cell responses in mice.^14^^,^^32^ Here, we show that UC patients in remission and healthy subjects differ in their fecal metabolomic profile, consistent with previous data.^17^ Without specific blocking experiments it is impossible to define which metabolites, or other molecules, are of importance for imprinting macrophages. Nonetheless, α-ketoglutaric acid has been shown to promote metabolic changes resulting in alternatively activated macrophages.^18^ Importantly, fumaric acid and succinic acid promote proinflammatory signaling but are both counteracted by α-ketoglutaric acid.^18^^,^^36^ Microbiota-derived SCFAs, which tend to be reduced in IBD patients with active disease,^37^^,^^38^ have been reported to induce anti-inflammatory properties on intestinal macrophages.^14^^,^^15^ In our study, healthy controls and UC patients had similar levels of SCFAs, but we cannot rule out differences in SCFA consumption of the epithelial layer and possibly of TNFα reducing butyrate uptake.^39^ Besides SCFAs, several microbiota-derived/modified metabolites with immunomodulating activities including indole derivatives, polyamines, and bile acids, could influence macrophage differentiation.^40^ In addition, bacterial particles, lipids, proteins, and nonmicrobial compounds such as antibodies^41^ that are altered during IBD, can be involved.

We acknowledge several limitations of the current study. First, the sample size was small and further validation with a larger cohort is required. Second, this was an in vitro system and the effects seen on monocyte-derived macrophages most likely differ from LP macrophages or in the presence of other tissue-derived signals. In addition, it remains to be clarified whether the FS concentrations and treatment durations used are physiologically relevant. Third, the fact that UC patients suffer from diarrhea, or in some cases constipation, during flare-ups may bring out the need for normalization of the FS. However, this is unlikely to be a major issue because the current study focuses on UC patients in remission with normal stool consistency. In addition, by studying UC patients in remission, we minimize the effects of factors associated with active inflammation, which could affect macrophage polarization (eg, cytokines).^42^ Finally, although *E coli* LPS was used to stimulate macrophages, it is known that there is a diverse pool of LPS in the human gut, where *E coli* LPS makes up only a minority of the total LPS.^43^

To conclude, fecal luminal factors derived from UC patients in remission fail to regulate TLR signaling and thereby induce LPS hyporesponsiveness in macrophages. Furthermore, UC remission FS-treated macrophages have lost their efficacy in suppressing CD4^+^ T-cell activation and cytokine secretion. Together with the distinct fecal metabolomic profile of UC patients in remission, our data suggest that UC patients may lack the signals required for proper macrophage education, rendering them vulnerable to relapse. Identification of the factors involved in intestinal macrophage education is important to maintain/re-establish gut homeostasis in patients with UC.

## Methods

### Study Samples and Ethical Considerations

UC patients and healthy subjects were included in the study, all were nonsmokers and older than 18 years of age. The UC patients (Table 3) were derived from 2 study cohorts recruited from 5 gastroenterology units in Western Sweden. Fecal samples were collected at 2 time points for the main cohort (1 time point during remission, and 1 time point during a flare) and from 1 time point during remission for the second cohort. The main cohort (used throughout this article) originally was recruited for a pharmacologic intervention study in UC patients on maintenance treatment with oral mesalamine (Asacol, Tillotts Pharma, Rheinfelden, Switzerland; Pentasa, Ferring, Saint-Prex, Switzerland; or Colazid, Almirall, Bacelona, Spain).^44^ UC patients were in remission at inclusion (total Mayo score, ≤2^45^) and were asked to provide monthly stool samples. A relapse was defined by colonoscopy or by an increase in UC symptoms and a calprotectin level greater than 300 μg/g. Using selection criteria of 1 stool sample during remission close to a flare (2–4 months before or after the flare) and 1 stool sample at the flare, 11 patients were included in this study. The second cohort (only used for Figure 2*B*) was derived from a 10-year follow-up period of an inception cohort.^46^ The selection criteria were 1 stool sample at remission with 1–2 flares during the previous year (n = 6). Exclusion criteria for both cohorts were ongoing anti-TNF therapy, corticosteroids or nonsteroidal anti-inflammatory drugs, antibiotics in the past month, pregnancy, prior colon resection, and comorbid diseases that would interfere with the study protocol.

**Table 3:** Demographic and Clinical Profiles of UC Patients

	Main cohort (n = 11)	Second cohort (n = 6)	*P*
Sex, male/female	5/6	3/3	.86^a^
Age, *y*	38 (21–58)^b^	40 (29–50)	.86^c^
Disease duration, *y*	5 (1–16)	10 (9–11)	.11^c^
Disease extent (proctitis/left sided/extensive)	0/9/2	1/1/4	.03^a^
Fecal calprotectin level, *mg/kg*			
Remission	49 (11–197)	55 (15–115)	.80^c^
Flare	860 (167–2312)	–	–
Treatment (mesalamine compounds/oral steroids/thiopurines)			
Remission	11/0/2	6/0/0	.31^a^
Flare	11^d^/4/2	–	–

a Chi -square test.

b Continuous data are shown as the median (range).

c Mann–Whitney *U* test.

d Increased mesalamine during a flare.

Healthy subjects (n = 10) were recruited from Sahlgrenska University Hospital (Gothenburg, Sweden).^47^ Here, 1 fecal sample was collected from each healthy subject (fecal calprotectin levels <30 mg/kg). Healthy subjects had a male-to-female ratio of 5:5 and a median age of 23.5 years (range, 20–36 y). Standardized questionnaires confirmed that none of the healthy subjects had any gastrointestinal complaints during the last week before inclusion. Furthermore, none of the healthy subjects had taken any immunosuppressive agents, antibiotics, or any other medication during the 3 months before inclusion.

All fecal samples provided were stored in the short term at -20°C, and then stored long term at -80ºC, and thawed only once.

### Ethical Statement

All study subjects provided written informed consent according to the Declaration of Helsinki. This study was approved by the Regional Ethical Review Board at the University of Gothenburg (Dnr 154-08 and 563-02).

### Preparation of Fecal Supernatants

FS were prepared by adding 2 weight volumes of phosphate-buffered saline to feces followed by homogenization into a suspension and centrifugation at 40,000*g* for 2 hours at 4°C. The supernatants were collected and filtered through a Costar Spin-X 0.22-μm cellulose acetate centrifuge tube filter (Corning, Inc, Corning, NY). FS aliquots were stored at -80°C until use.

### CD14^+^ and CD4^+^ Blood Cell Isolation

Peripheral blood mononuclear cells were isolated from buffy coats obtained from healthy donors (Clinical Immunology and Transfusion Medicine, Sahlgrenska University Hospital, Gothenburg, Sweden; permit K 15/18) using Ficoll-Hypaque (GE Healthcare, Chicago, IL). CD14^+^ monocytes were isolated from peripheral blood mononuclear cells using CD14 microbeads and CD4^+^ T cells using the CD4^+^ T-Cell Isolation Kit (both from Miltenyi Biotech, Bergisch Gladbach, Germany). The CD4^+^ T cells were immediately frozen in N_2_ after purification. All procedures were performed according to the manufacturers’ protocols.

### M1 Macrophage Differentiation and FS Treatment

Isolated CD14^+^ cells were suspended in serum-free base media containing 1× GM-CSF using the CellXVivo Human M1 Differentiation Kit (R&D Systems, Minneapolis, MN) and 50 μg/mL gentamicin (Gibco, Waltham, MA). The cells were cultivated in Nunc MicroWell 96-Well Microplates (Thermo-Fischer, Waltham, MA) at 2 × 10^5^ cells/well in 200 μL media and incubated at 37°C, 5% CO_2_. After 3 days, half of the media was replaced with fresh serum-free base media with 1 × GM-CSF, with or without FS at different dilutions. The cells were incubated for an additional 3 days. On day 6, the media was removed and the differentiated cells were used for further analyses. To detach the macrophages for functional analyses, Hanks' balanced salt solution and Cell Dissociation Solution (both Gibco) were preheated to 37°C. Then the cells were washed with Hanks' balanced salt solution, Cell Dissociation Solution was added to the wells and removed after 30–60 seconds, and the plates were incubated for 10 minutes at 37°C, 5% CO_2_. After incubation, media was added to the wells and the cells were loosened.

### TLR Stimulation of M1 Macrophages

After differentiation and treatment with FS, the cells were stimulated with TLR ligands in complete media; Iscove’s media (Merck, Kenilworth, NJ) supplemented with 10% fetal bovine serum (Biological Industries, Beit HaEmek, Israel), 200 mmol/L L-glutamine (Sigma, St. Louis, MO), and 50 μg/mL gentamicin. For stimulation, complete media containing 10 ng/mL LPS (LPS-EK), 10 ug/mL PGN (PGN-BS), 50 ng/mL flagellin (FLA-ST), or 5 umol/L CpG oligonucleotides (ODN2006) was added (all Invivogen). After 24 hours of stimulation, the supernatants were collected and stored at -20°C. The cells were collected and used either for flow cytometry or for quantitative PCR analysis.

### Enzyme-Linked Immunosorbent Assay

The concentration of TNFα, IL1β, IL10, CXCL10, IL17, and IFNγ in the cell supernatants was measured using an enzyme-linked immunosorbent assay (ELISA) according to the manufacturers’ protocols (Human TNFα Uncoated ELISA, eBioscience, San Diego, CA; Human IL1β Uncoated ELISA, eBioscience; ELISA MAX Deluxe Set Human IL10, BioLegend, San Diego, CA; ELISA MAX Deluxe Set Human CXCL10 (IP-10), BioLegend; Human IL17A alpha Uncoated ELISA, Invitrogen; Human IFN gamma Uncoated ELISA, Invitrogen, Waltham, MA). LPS concentration in the FS samples was assessed by the LPS ELISA Kit (Aviva Systems Biology, San Diego, CA). The concentration of calprotectin in fecal samples was determined by a sandwich ELISA (Calprotectin ELISA; Bühlmann Laboratories AG, Basel, Switzerland).

### Fluorescence-Activated Cell Sorting Analysis

Cells were stained with antibodies at 4°C, in the dark, for 20–30 minutes. Different combinations of the following antibodies were used: anti-CD3 (fluorescein isothiocyanate), anti-CD14 (BUV737), anti-CD64 (allophycocyanin), anti-HLA-DR (BUV395), anti-CD80 (BV421), anti-CD25 (BV421), and anti-CD4 (phycoerythrin-Cy7) (all from BD Biosciences, Franklin Lakes, NJ). Dead cells were excluded using 7-amino actinomycin D (BD Biosciences). Cells were acquired on a Fortessa X20 (BD Biosciences) and the data were analyzed using FlowJo software (Tree Star, Ashland, OR).

### RNA Extraction and Gene Expression Profiling

Total RNA was extracted using the NucleoSpin RNA kit (Macherey-Nagel GmbH, Düren, Germany). The purity and quantity of RNA was assessed using a NanoDrop ND-1000 spectrophotometer (NanoDrop Technologies, Wilmington, DE) with 260/280 and 260/230 ratios of approximately 2 and 2.1–2.2, respectively.

Myeloid innate immune response–related gene expression was examined using the nCounter Human Myeloid Innate Immunity Panel v2 (NanoString Technologies, Inc) at the Karolinska Institute KIGene Core Facility according to the manufacturer’s instructions. The panel included 770 transcripts associated with 19 different pathways and processes. Briefly, isolated RNA (100 ng) was hybridized with proprietary capture and reporter probes. The RNA-probe complexes were purified, immobilized, and counted. The obtained raw data were normalized against internal controls and housekeeping genes (selected by the built-in geNorm algorithm) using the nCounter Advanced Analysis software v2 (NanoString Technologies).

The expression of TLR signaling–associated genes (84 genes) was profiled by the RT^2^ Profiler PCR Array Human Toll-Like Receptor Signaling Pathway (PAHS-018Z; Qiagen, Hilden, Germany) according to the manufacturer’s instructions. Before PCR analysis, the isolated RNA was converted to complementary DNA using the Quantitect Reverse Transcription Kit according to the manufacturer’s protocol (Qiagen, Hilden, Germany). PCR was performed using the 7500 Real Time PCR system (Applied Biosystems, Waltham, MA). Three of 84 genes were excluded from the TLR array analysis owing to missing data in >50% of the samples (*CSF2*, *IFNA1*, and *IL2*). *ACTB*, *B2M*, *GAPDH*, *HPRT1*, and *RPLP0* were used as housekeeping genes. Gene expression was calculated by the 2^−ΔCT^ method. Fold change was calculated by comparing the gene expression of the FS-treated cells (UC and healthy) with FS-untreated cells (control) using the 2^−ΔΔCT^ method. All samples passed the quality checks for PCR array reproducibility, reverse transcription efficiency, and genomic DNA contamination.

### Phagocytosis Assay

Macrophages were suspended in complete media containing 10 ng/mL LPS, cultivated at 10,000 cells/well in clear-bottom, black, 384-well plates (Greiner Bio-One, Kremsmünster, Austria), and incubated overnight. Next, cells were incubated with fluorescent carboxylated beads (Polysciences, Hirschberg an der Bergstraße, Germany) or LPS-coated beads (carboxylated beads coated with LPS) at 1:100 dilution for 30 minutes. Nonphagocytosed beads were quenched by undiluted trypan blue. The fluorescence was measured using the SpectraMax i3x luminescence multimode microplate reader at an excitation/wavelength of 441/486 nm (Molecular Devices, San Jose, CA).

### Bacterial Killing Assay

Macrophages were suspended in complete Iscove’s media without gentamicin at 50,000 cells/well in 96-well plates and incubated overnight at 37ºC, 5% CO_2_. The bacterial killing ability of cells was measured using the gentamicin protection assay.^48^ *E coli* HS and HM427 were grown in Luria Broth at 37°C, shaking at 200 rpm until OD600 reached 1.0–1.5. The concentration of bacteria was adjusted using complete Iscove’s media without gentamicin (OD600 1.0 ≈ 5 × 10^8^ bacteria). Bacterial suspension was added to the cells with a multiplicity of infection of 1:10 (cell:bacteria). Two identical plates were prepared in parallel, named T15 and T120. The plates were centrifuged at 500*g* for 5 minutes, at room temperature, to increase the contact between bacteria and macrophages. The plates were incubated at 37°C, 5% CO_2_, for 30 minutes to let the cells phagocytose the bacteria. After the incubation, the supernatant was aspirated and replaced with Iscove’s media containing 0.5% gentamicin. The T15 and T120 plates were incubated further for 15 minutes and 120 minutes, respectively. To lyse the cells, the supernatants were removed and 1% saponin diluted in H_2_O was added to the wells. The plates were incubated on ice for 10 minutes. The lysed cells were diluted using H_2_O and plated on Luria broth plates, incubated at 37°C overnight, and the colonies were counted. The killing percentage was calculated as follows: ([T15-T120]/T15) ∗ 100. The *E coli* HS and HM427 were provided by Åsa Keita (Linköping University, Linköping, Sweden).

### Autologous Mixed Lymphocyte Reaction

Macrophages were suspended in complete media containing 100 ng/mL LPS, seeded at 20,000 cells/well in a 96-Well Round (U) Bottom Plate (Thermo-Fischer), and incubated overnight at 37ºC, 5% CO_2_. CD4^+^ T cells obtained from the same donor as the monocytes were thawed, stained with 5, 6-carboxyfluorescein diacetate succinimidyl ester (Invitrogen, Carlsbad, CA), and co-incubated with the macrophages at a ratio of 1:1 in the presence of 1 μg/mL anti-CD3 monoclonal antibody (BD Pharmingen, San Diego, CA) for 3 days. Supernatants were collected and the cells were analyzed by flow cytometry.

### Metabolomic and SCFA Analyses

The metabolomic and SCFA profiles of fecal supernatant samples were analyzed at Chalmers Mass Spectrometry infrastructure (Chalmers University of Technology, Gothenburg, Sweden). Metabolites were analyzed using gas chromatography coupled to a tandem mass spectrometer (GC-MS/MS). Briefly, the fecal supernatants were extracted with a mixture of water and methanol containing 10 stable isotope-labeled internal standards,^49^ followed by drying and derivatization using oxymation and silylation. Derivatized extracts were injected into a GC-MS/MS system (Shimadzu Europa GmbH, Duisberg, Germany) and GC-MS scan data (50–750 m/z) were analyzed for targeted peak detection. Peaks were identified based on a Matlab script and data were normalized based on the internal standard peak intensities.^50^ The concentrations of SCFA were determined by capillary gas GC by a method developed by Richardson et al.^51^ This method detects acetic acid, propionic acid, butyric acid, isobutyric acid, valeric acid, isovaleric acid, and caproic acid. Briefly, samples were diluted with distilled water (1/4), and 2-ethylbutyric acid (5 mmol/L) was added as internal standard. Samples then were extracted in diethyl ether, derivatized with N-tert-butyldimethylsilyl-N-methyltrifluoroacetamide, and analyzed on Agilent GC HP-1 capillary columns (Agilent Technologies, Santa Clara, CA).

### Data and Statistical Analyses

Data and statistical analyses were performed using R 4.0.2 and GraphPad Prism 8.0.2 (GraphPad Software, San Diego. CA). The level for significance was set to 0.05. PCAs for gene expression and metabolite data were performed using the pca3d package in R, after log_10_ transformation of the data. PCA analysis of the T-cell data was performed using the FactoMineR and factoextra packages in R and show the mean from 2 separate experiments with identical set-up of FS samples. Because of variability based on the donor of monocytes and T cells, the results were first z-normalized before calculating the mean values for the 2 experiments. The 2-sample Hotelling’s T2 test was used to test differences between 2 groups for multiple parameters using the R Hotelling package. Volcano plots for the NanoString data were obtained using nCounter Advanced Analysis software v2 (NanoString Technologies). Volcano plots for the TLR array and metabolite data were generated using the ggplot2 and ggrepel packages in R, the log_2_ fold change of the means was calculated and differences between groups were determined using the Student *t* test. For all other tests between 2 unrelated groups, the Mann–Whitney *U* test was used. The Wilcoxon test was used for testing differences between 2 related samples. The Kruskal–Wallis test followed by the Dunn multiple comparisons test was used for analysis of 3 independent groups. To analyze the association between 2 parameters, Pearson correlation was used. The chi-square test was used to analyze the differences between categoric variables. The Benjamini–Yekutieli method was applied to adjust the *P* values for multiple comparisons. Pathway analysis for the metabolite data was performed using MetaboAnalyst 4.0 online website (https://www.metaboanalyst.ca).^52^

All authors had access to the study data and had reviewed and approved the final manuscript.

## CRediT Authorship Contributions

Lujain Maasfeh, MSc (Conceptualization: Supporting; Formal analysis: Lead; Investigation: Equal; Methodology: Equal; Writing – original draft: Lead; Writing – review & editing: Equal)

Anetta Hartlova, PhD (Formal analysis: Supporting; Funding acquisition: Supporting; Methodology: Supporting; Writing – review & editing: Equal)

Stefan Isaksson, BSc (Conceptualization: Supporting; Formal analysis: Supporting; Methodology: Supporting; Supervision: Supporting; Writing – review & editing: Equal)

Johanna Sundin, PhD (Investigation: Supporting; Resources: Equal; Writing – review & editing: Equal)

Georgios Mavroudis, MD PhD (Investigation: Supporting; Resources: Equal; Writing – review & editing: Equal)

Otto Savolainen, PhD (Formal analysis: Equal; Investigation: Supporting; Methodology: Supporting; Writing – review & editing: Equal)

Hans Strid, MD PhD (Conceptualization: Supporting; Investigation: Equal; Resources: Equal; Writing – review & editing: Equal)

Lena Öhman, Professor (Conceptualization: Equal; Funding acquisition: Lead; Resources: Equal; Writing – original draft: Supporting; Writing – review & editing: Equal)

Maria K Magnusson, PhD (Conceptualization: Lead; Formal analysis: Supporting; Funding acquisition: Equal; Investigation: Equal; Methodology: Equal; Software: Lead; Supervision: Lead; Writing – original draft: Supporting; Writing – review & editing: Equal)
